# HSP27 phosphorylation inhibitor regulates Her2 expression in human breast cancer cell line SK-BR-3 with induced Herceptin resistance

**DOI:** 10.1186/1878-5085-5-S1-A47

**Published:** 2014-02-11

**Authors:** Min Kyung Kim, Seung Cheol Kim, Won Ki Kim, Kun Kim, Kyung-Hee Kim, Byong Chul Yoo

**Affiliations:** 1 Colorectal Cancer Branch, Division of Translational and Clinical Research I, Research Institute, National Cancer Center, Gyeonggi, 410-769, Republic of Korea; 2 Division of Gynecologic Oncology, Department of Obstetrics and Gynecology, Ewha Womans University Mokdong Hospital, College of Medicine, Ewha Womans University Seoul, 158-710, Republic of Korea

## 

Herceptin, HER2-targeted monoclonal antibody, is one of the main treatments for breast cancer. However, the majority of patients that initially respond to Herceptin begin to progress again within 1 year. Thus, it is very important to clarify how to overcome resistance as much as to identify breast cancer patients who will be benefit from Herceptin. We previously reported that HSP27 can interact with Her2 protein, and up-regulated HSP27 may cause Herceptin resistance by increasing Her2 protein stability in human breast cancer cell line SK-BR-3 with induced Herceptin resistance (SK-BR-3 HR). In the present study we investigated whether HSP27 phosphorylation inhibitor can reduce the resistance of Herceptin by breaking interaction between Her2 and HSP27 protein. HSP27 phosphorylation inhibitor not only suppressed cell proliferation but also augmented apoptotic cell death in both SK-BR-3 HR and its parent cell line. Such anti-cancer effects were much stronger in SK-BR-3 HR compared to its parent cell line, and regulation of Her2 after HSP27 phosphorylation inhibitor treatment was found only in SK-BR-3 HR. However, the interaction between HSP27 and Her2 was not broken, but became increased by HSP27 phosphorylation inhibitor in a concentration dependent manner. Overall results demonstrate that even HSP27 phosphorylation inhibitor does not directly target the interaction of HSP27 with Her2; it would be useful to alternative option to overcome Herceptin resistance because it may induce effective down-regulation of Her2.

